# Macroautophagy in sporadic and the genetic form of Parkinson's disease with the A53T α-synuclein mutation

**DOI:** 10.1186/2047-9158-1-2

**Published:** 2012-01-13

**Authors:** Yue Huang, Fariba Chegini, Germaine Chua, Karen Murphy, Weiping Gai, Glenda M Halliday

**Affiliations:** 1 Neuroscience Research Australia and the University of New South Wales, Sydney, 2031, Australia; 2 Department of Human Physiology and the Centre for Neuroscience, Flinders University School of Medicine, Bedford Park, SA5042, Australia

**Keywords:** α-synuclein, macroautophagy, Parkinson's disease

## Abstract

**Background:**

The A53T mutation in the *α-synuclein *gene causes autosomal-dominant Lewy body Parkinson's disease (PD). Cultured cell models have linked this mutation to increased cell macroautophagy, although evidence of enhanced macroautophagy in patients with this mutation has not been assessed.

**Objective:**

To determine whether macroautophagy is increased by the A53T *α-synuclein *gene mutation in PD patients and cell models.

**Methods:**

Formalin-fixed paraffin-embedded 10 μm-thick tissue sections from the substantia nigra and anterior cingulate cortex of two PD patients with the A53T *α-synuclein *gene mutation were compared with four sporadic PD cases and four controls obtained from the Sydney Brain Bank. Lewy bodies were isolated from frontal cortex of a case with late stage PD (recruited from South Australian Brain Bank). Immunohistochemistry was performed for α-synuclein and the macroautophagy markers autophagy-specific gene (ATG) 5, ATG6/Beclin1 and ATG8/LC3. SH-SY5Y cells were transfected with wild type or A53T mutant *α-synuclein *plasmids and observable changes in macroautophagy marker protein levels assessed using Western blotting.

**Results:**

α-Synuclein immunoreactive neurites and dots were more numerous in patients with A53T mutations compared with late stage sporadic PD patients, and perinuclear cytoplasmic α-synuclein aggregates were observed in the *α-synuclein *A53T gene transfected SH-SY5Y cells compared to wild type transfections. All PD patients (with or without A53T mutations) had increased immunohistochemical evidence for macroautophagy compared with controls, and the levels of the ATG5 complex were equally increased in wild type and A53T *α-synuclein *gene transfected cells compared to controls.

**Conclusion:**

Despite increased α-synuclein accumulation with A53T mutations, macroautophagy is not increased above that observed in sporadic patients with PD or in cells transfected with wild type α-synuclein, suggesting that mutated α-synuclein protein is not removed by macroautophagy.

## Introduction

Genetic forms of Parkinson's disease (PD), the most common neurodegenerative movement disorder affecting the elderly, provide important information on major cellular abnormalities occurring in sporadic PD and are now used in relevant models to understand disease pathogenesis. Missense mutations in (A53T [1], A30P [2], E46K [3]) or multiplications of [4] the *α-synuclein *gene [5,6] occur only rarely in PD patients, however abnormalities in the cellular processing of α-synuclein are considered core to PD pathogenesis due to its abnormal deposition and fibrilisation within the characteristic pathological inclusions of PD [7,8].

The formation of cytoplasmic α-synuclein pathologies in PD has been attributed to the dysfunction of protein degradation pathways through the proteasome [9] and/or lysosomal autophagy [10,11]. The lysosome participates in three types of autophagy: macroautophagy, microautophagy (lysosomes directly engulf cytoplasmic contents) and chaperone-mediated autophagy (CMA, receptor-specific recognition for protein transfer into lysosomes). Macroautophagy sequesters damaged organelles and unused long-lived proteins in a specialised autophagic vacuole or autophagosome, which then fuses with a lysosome. Macroautophagy is the most efficient autophagic clearance mechanism and the most important for organelle clearance in neurons [12].

Autophagy-specific gene (ATG) proteins are used to assemble autophagosomes within cells. ATG6/Beclin 1 binds with the anti-apoptotic protein Bcl-2 located in the cytoplasm, and its dislocation from Bcl-2 is essential for the initiation of autophagosome generation [13]. ATG5 complexes with ATG12, which then interacts with lipidated ATG8/LC3, indicating autophagic activation [14]. These processes have been shown to be upregulated in neurons undergoing neurofibrillary degeneration in Alzheimer's disease [14]. In contrast, dysfunctional autophagosomes and reduced ATG7 but preservation of many other macroautophagy markers has been described in association with α-synuclein deposition in dementia with Lewy bodies [15]. In cultured neurons, A53T mutant α-synuclein protein enhances macroautophagy [16,17], but this has not been examined in patients with PD.

## Materials and methods

### Cases

Formalin-fixed paraffin-embedded 10 μm-thick sections of midbrain and anterior cingulate cortex (an early affected limbic brain region [7,8]) of two PD patients with the A53T *α-synuclein *gene mutation, four sporadic PD patients and four controls were obtained from the Sydney Brain Bank following appropriate institutional approvals (case details are given in Table 1). Similarly processed sections from the hippocampus of a case with Alzheimer's disease were included as positive controls for the immunohistochemistry [14]. One PD case with Braak stage 6 was recruited from the South Australian Brain Bank for the isolation of cortical Lewy bodies, as previously described [18].

**Table 1:** Details of the cases examined for morphological observations

Diagnosis	**Case No**.	Sex (M/F)	AOD(y)	PMD(hr)	Braak PD stage (0-6)	Density of α-synuclein inclusions in cingulate ctx	Density of ATG8/LC3 immunostaining in cingulate ctx
Control	1	F	64	5	0	None	None
	
	2	M	68	11	0	None	None
	
	3	M	75	<24	0	None	None
	
	4	F	81	17	0	None	None

Sporadic PD	1	F	69	<24	4	None	None
	
	2	M	72	<36	5	Mild	None
	
	3	M	79	3	5	Moderate	Mild
	
	4	F	78	4	6	Severe	Moderate

α-*Synuclein*	1	M	48	27	6	Severe	Moderate
A53T PD	2	M	54	<24	6	Severe	Moderate

ctx = cortex; No. = Number; AOD = age of death; PMD = post-mortem delay

### Cell culture and α-synuclein plasmid transfections

SH-SY5Y cells were obtained from ATCC, USA and cultured at 37°C in Dulbecco's modified eagle media supplemented with 10% fetal bovine serum, 100 U/ml of penicillin and 100 μg/ml of streptomycin in a humidified 5% CO_2 _incubator. Cells were transfected using Lipofectamine 2000 with plasmids (kind gifts from P.J. McLean) expressing GFP-tagged wildtype (WT) *α-synuclein *or GFP-tagged A53T mutant (A53T) *α-synuclein*. Cells exposed to Lipofectamine 2000 only were used as controls. The transfection rate, cellular morphology and the amount of proteins of interest were observed at 24 h.

### Western blotting

SH-SY5Y cells expressing WT and A53T α-synuclein were harvested and solubilised with 2X SDS sample buffer (20 mM dithiothreitol, 6% SDS, 0.25 M Tris, pH 6.8, 10% glycerol, 10mMNaF and bromophenol blue) at approximately 2 × 10^6^-1 × 10^7 ^cells per ml. The extracts were heated in a boiling water bath for 5 min, and then sonicated with 3-4 bursts of 5-10 sec each, and finally separated using 4-12.5% SDS-PAGE. Western blotting was performed using mouse anti-human α-synuclein IgG (ab6162, Abcam, USA, diluted 1 mg/ml), rabbit anti-human ATG5 (Abgent, USA, diluted 1:100), rabbit anti-human ATG6/Beclin 1 (Abgent, USA, diluted 1:60) and rabbit anti-human ATG8/LC3 (Abgent, USA, diluted 1:1000). Mouse anti-human β-tubulin III (ab7751, Abcam, USA, diluted 1:1000) was used to assess equal protein loading. Experiments were repeated three times.

### Immunohistochemistry

Peroxidase immunohistochemistry for α-synuclein (mouse anti-human α-synuclein, BD Transduction Laboratories, Lexington, USA, diluted 1:200) and the macroautophagy markers ATG5 (rabbit anti-human ATG5, Abgent, USA, diluted 1:50), ATG6/Beclin 1 (rabbit anti-human ATG6, Abgent, USA, diluted 1:10) and ATG8/LC3 (rabbit anti-human ATG8, Abgent, USA, diluted 1:1000) was performed on the human brain tissue sections (Table 2). Briefly, sections were pre-treated with 99% formic acid for 3 min and citrate buffer (pH 6.0) for 3 min, and then following primary antibody incubation, incubated with biotinylated secondary antibodies (Vector, Burlinghame, USA), avidin-biotin complex (Vectastain Elite ABC Kit, Vector, Burlinghame, USA) and DAB substrate (Sigma, St. Louis, USA) then counterstained with cresyl violet.

**Table 2:** Macroautophagy Markers Tested and Their Dilutions

Macroautophagy Markers	Human Brain Tissues (Immunohistochemistry)	Cultured Cells (Western blotting)
Atg 5	1:50	1:100

Atg 6/Beclin 1	1:10	1:60

Atg 8/LC3	1:1000	1:1000

Co-localisation of the macroautophagy markers with α-synuclein was assessed on adjacent human brain tissue sections, isolated Lewy bodies, as well as on SH-SY5Y cells expressing WT and A53T *α-synuclein *that were fixed using methanol-acetone (3:1) for 15 min. Briefly, double labelling immunofluorescence was performed with secondary anti-mouse IgG conjugated with Alexa Fluor 488 (1:500, Molecular Probes, Oregon, USA) and anti-rabbit IgG conjugated with Alexa Fluor 568 (1:250, Molecular Probes). The cross-reactivity and specificity of the immunofluorescence was confirmed by incubating each primary antibody singly with the secondary antibody solution containing two fluorophores. Experiments were repeated three times.

### Morphological observations

α-Synuclein and macroautophagy markers immunoreactive aggregates and cells were semi-quantified as mild, moderate or severe in the immunoperoxidase-labeled brain tissue sections, and the proportion of transfected SH-SY5Y cells containing α-synuclein-immunopositive aggregates was counted in five representative fields from each experiment. The co-localisation of α-synuclein and macroautophagy markers was examined at 100×magnification using a confocal microscope (Leica Microsystems Heidelberg GmbH, Germany).

## Results

### Differences in the amount and type of α-synuclein aggregates in A53T versus sporadic PD

**Figure 1:**
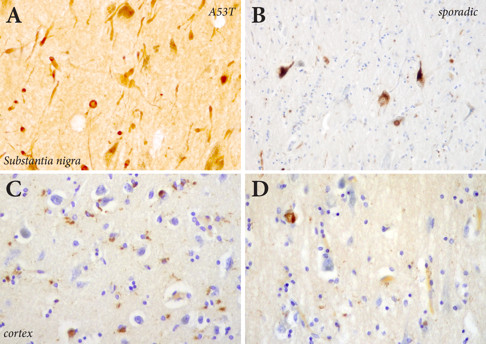
**α-Synuclein immunohistochemistry in PD**. Although both sporadic cases and those with A53T *α-synuclein *mutations had severe neuronal loss in the substantia nigra, substantially more α-synuclein positive neurites and dots were seen in the A53T cases (**A**) compared with sporadic PD (**B**). As expected, there were more α-synuclein-immunopositive Lewy body and astrocytes in the cortex of end-stage (**C**) versus earlier stages of PD (**D**).

Both A53T and sporadic PD had moderate to severe loss of substantia nigra neurons, as previously described [19]. There were significantly more α-synuclein-immunoreactive Lewy neurites (Figure 1A) in the A53T form of PD compared with sporadic PD (Figure 1B) [19]. These neurites were substantially larger in cases with A53T mutations (Figure 1A). As expected, there were more α-synuclein-immunoreactive cortical Lewy bodies and astrocytes in end-stage (Figure 1C) compared with earlier-stages of PD (Figure 1D). The relative densities of α-synuclein-immunoreactive cortical Lewy bodies are given in Table 1.

### Accumulation and localisation of macroautophagy markers in A53T versus sporadic PD

**Figure 2:**
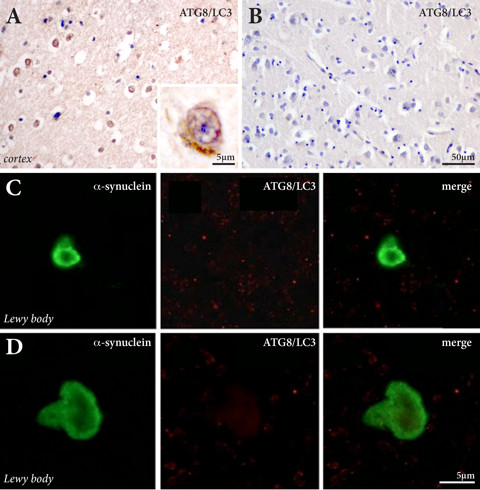
**Macroautophagy marker immunohistochemistry in PD**. (**A**) ATG8/LC3-immunopositive cells were observed in both end-stage sporadic PD as well as in cases with A53T mutations where α-synuclein accumulations are found in the cortex, and were not observed in earlier PD stages or in controls (**B**) where there is an absence of cortical α-synuclein accumulation. In isolated Lewy bodies identified with α-synuclein antibodies (**C, D**), there was no ATG8/LC3immunoreactivity.

Consistent with previous studies in dementia with Lewy bodies [15], there was an increase in ATG8/LC3-immunopositive cortical neurons with increasing pathological stage of PD, and some glia also had enhanced ATG8/LC3 expression (Figure 2A). The density of ATG8/LC3-immunopositive cortical neurons did not differ between the cases with A53T mutations and those with end-stage sporadic PD (Figure 2A & Table 1), both of which had substantial cortical deposition of α-synuclein (see above). In cases with no cortical deposition of α-synuclein (stage 4 PD and controls), no cortical ATG8/LC3 immunoreactive neurons were observed (Figure 2B). Double labeling of isolated Lewy bodies with antibodies to α-synuclein and ATG8/LC3 also showed no colocalisation (Figure 2C, D). Immunohistochemistry for the other macroautophagy markers assessed was not significantly increased over controls in any of the PD case assessed (data not shown), similar to that described previously for cases with dementia with Lewy bodies [15].

### Macroautophagy following overexpression of WT or A53T α-synuclein in SH-SY5Y cells

**Figure 3:**
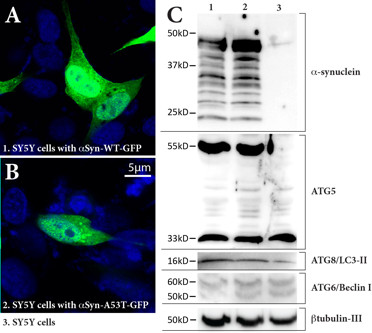
**Macroautophagy markers in SH-SY5Y cells overexpressing wild-type (WT) or A53T mutant *α-synuclein***. **A**. α-Synuclein-immunopositive dots and homogenous staining of α-synuclein in SH-SY5Y cells over-expressing WT *α-synuclein *plasmid transfection. **B**. Larger α-synuclein-immunopositive aggregates in SH-SY5Y cells with A53T *α-synuclein *plasmid transfection. **C**. Truncated small α-synuclein fractions were detected in both WT (lane 1) and A53T mutant (lane 2) *α-synuclein *transfected cells (20~49kDa) (non-transfected cells in lane 3). The ATG5-ATG12 complex (55kDa) was significantly increased in cells overexpressing α- synuclein protein (GFP-fused major band at ~49kDa, lanes 1 and 2). Repeat experiments showed there were no observable differences between cells transfected with WT (lane 1) or mutant (lane 2) *α-synuclein *in the levels of ATG5 monomer (band at ~33kDa), the active form of ATG8/LC3-II (~16kDa), or inATG6/Beclin1 (~60kDa), under the condition of equal protein loading (internal control of β-tublin-III at ~50kDa).

Approximately 60% of the SH-SY5Y cells transfected with A53T *α-synuclein-GFP *plasmids contained α-synuclein-immunopositive aggregates (Figure 3A) compared with approximately 30% of those transfected with WT *α-synuclein-GFP *plasmids (Figure 3B). These aggregates did not colocalise with macroautophagy markers (data not shown). The α-synuclein-immunopositive aggregates in cells transfected with A53T *α-synuclein-GFP *plasmids (Figure 3A) were much larger compared to those in cells transfected with WT *α-synuclein-GFP *plasmid (Figure 3B). In addition, transfection with A53T *α-synuclein *significantly reduced cell viability in comparison to WT *α-synuclein *transfection (data not shown). When the same amount of cellular protein was loaded, there were similar multiple 20~49kDa bands of GFP-tagged α-synuclein detected in both WT and A53T transfected cells (Figure 3C), as previously reported [9]. Western immunoblotting revealed a significant increase in the macroautophagy marker for the ATG5 complex in both types of cells overexpressing *α-synuclein *compared to controls (Figure 3C). Such an increase is not due to transfection reagent exposure, given the presence of ATG8/LC3-II in control SH-SY5Y cells (Figure 3C). LC3-II (phosphatidyl ethanolamine-modified form of LC3) is stably associated with autophagosomal membranes and is therefore considered an autophagy-specific marker [20]. In addition to the full length 60kDa ATG6/Beclin 1 [21], its 50kDa cleavage proteolytic fragment appears in all three groups of cells. There is no significant difference between the observable levels of LC3-II and ATG/Beclin 1 in cells transfected with different *α-synuclein *plasmids and controls.

## Discussion

We observed increased α-synuclein-immunoreactive neurites in PD cases with A53T mutations, as previously described [22]. Clinically, these cases have an earlier onset and shorter disease duration compared to sporadic PD. These changes are also seen in mice expressing A53T mutant α-synuclein but not WT α-synuclein, as the mutant-expressing mice develop age-dependent intracytoplasmic neuronal α-synuclein inclusions and severe motor impairment [23]. Our cell culture data showed reduced cell viability of the A53T α-synuclein mutant expressing cells compared to those with WT α-synuclein expression, consistent with previous reports [24]. We also observed a significant increase in the numbers of α-synuclein-immunoreactive aggregates in A53T compared to WT *α-synuclein *transfected SH-SY5Y cells, as previously described [24]. These data support previous findings that A53T mutant α-synuclein aggregates more rapidly in neurons, is degraded more slowly and has greater toxicity than WT α-synuclein [25]. Intracellular α-synuclein aggregation can be due to an increase in its expression, such as observed in cases of *α-synuclein *gene multiplication [4], or a decrease in its clearance through autophagic pathways [26-30]. Autophagy is an evolutionarily conserved process by which eukaryotic cells regulate the turnover of long-lived proteins and cytoplasmic organelles. As described in the introduction, there are three types of autophagy: macroautophagy, microautophagy and chaperone-mediated autophagy (CMA). WT, but not A53T mutant, α-synuclein is degraded through the CMA pathway [11]. However, A53T mutant or other modifications to WT α-synuclein, or the overexpression of WT α-synuclein, leads to CMA dysfunction in neurons, and this in turn leads to a compensatory induction of macroautophagy [31-33]. Therefore, we investigated changes in macroautophagy markers in PD patients with the A53T mutation and sporadic PD, and appropriate cell models.

We observed elevated expression of macroautophagy markers in cells overexpressing either WT or A53T mutant α-synuclein, and there was no significant difference in macroautophagy expression between cells transfected with WT or mutant *α-synuclein*, suggesting a compensatory induction of macroautophagy [31-33]. The induction of macroautophagy was not further enhanced by the more toxic A53T species of α-synuclein. Our study is the first to show that cellular α-synuclein overexpression significantly elevates the levels of the ATG5 complex, regardless of whether WT or mutant α-synuclein is expressed, consistent with enhanced macroautophagy. This contrasts with recent findings of a reduction in ATG8/LC3-II in WT *α-synuclein *transfected cells but not in A53T mutant *α-synuclein *transfected cells [34]. After standardising for the number of cells transfected, our data show only subtle changes at best between WT and A53T mutant *α-synuclein *transfected cells using a greater number of macroautophagy markers, findings consistent with our observations in PD brain tissue.

We observed an increase in ATG8/LC3-immunopositive cortical neurons in late-stage PD, consistent with previous findings in dementia with Lewy bodies [15,35]. We are the first group to investigate macroautophagy markers in PD cases with A53T mutations compared to sporadic PD. We observed no differences between the relative densities of ATG8/LC3-immunopositive neurons in end-stage sporadic PD cases and those with A53T mutations, despite greater densities of α-synuclein accumulations in the A53T mutation cases. These data show that macroautophagy is induced in association with α-synuclein aggregation, but this increase does not appear to be directly related to its WT or mutant forms.

Our data also show that macroautophagy markers do not co-localise with the large α-synuclein aggregates either in brain tissue or cultured cells. Their pattern of staining is more reminiscent of organelle macroautophagy. Macroautophagy of mitochondria (mitophagy) is now thought to be a significant contributor to many different forms of PD, including patients with mutations in PINK-1, parkin and most recently PARL [36,37]. In addition, cell culture models show that mutant A53T α-synuclein induces mitophagy [16] and that increased WT α-synuclein impairs mitochondrial function independently of cellular α-synuclein aggregation [38]. The cellular aggregation of α-synuclein has recently been linked to cytoprotective changes associated with decreased mitochondrial calcium [39]. These data are consistent with our findings that macroautophagy markers do not routinely engulf or accumulate with insoluble intracellular α-synuclein, possibly indicating a more important role for macroautophay in mitophagy in PD rather than in α-synuclein aggregate disposal. Overall, our data show a similar increase in the levels of macroautophagy in both sporadic PD and in cases with more toxic A53T mutations.

## Competing interests

The authors declare that they have no competing interests.

## Authors' contributions

YH participated in study design, data analysis and manuscript drafting. FG & WPG carried out cell culture & isolated Lewy Bodies studies. GC & KM carried out human brain immunohistochemistry study. GMH conceived & coordinated of the study, and critically revised the manuscript for submission. All authors read and approved the final manuscript.
